# Association between insufficient medication of antihypertensives and the severity of acute ischemic stroke

**DOI:** 10.1186/s40885-016-0047-8

**Published:** 2016-02-19

**Authors:** Kyung Bok Lee, Jeong-Yoon Lee, Nari Choi, Jee-Eun Yoon, Dong-Won Shin, Ji-Sun Kim, Hakjae Roh, Moo-Young Ahn, Hye-Won Hwang, Min-Su Hyon

**Affiliations:** 1 Departments of Neurology, Soonchunhyang University, College of Medicine, 59 Daesakwan-ro, Yongsan-gu, Seoul, 04401 Korea; 2 Departments of Cardiology, Soonchunhyang University, College of Medicine, Seoul, Korea

**Keywords:** Antihypertensive, Stroke severity, NIHSS, Hypertension, Adherence

## Abstract

**Background:**

Although recent studies have suggested that adherence to antihypertensive treatment reduced stroke incidence, the relationship of adherence to antihypertensives with stroke severity has not been studied. This study attempted to know whether nonadherence before stroke is associated with initial severity of acute ischemic stroke.

**Methods:**

Consecutive patients with acute ischemic stroke were identified in Soonchunhyang University Hospital from Mar 2005 to Aug 2014, excluding the cases without hypertension or information of antihypertensive adherence. We compared the mean of National Institute of Health Stroke Scale (NIHSS) score between adherence groups and insufficient medication group, and additionally in each stroke subtype. Multiple linear regression model was established for initial NIHSS score adjusting alleged factors linked to stroke severity.

**Results:**

Initial NIHSS score were higher in insufficient medication group than adherence group (6.5 ± 7.2 *VS* 5.4 ± 5.7, *P* = .11). In large artery atherosclerosis (LAA) and small vessel occlusion (SVO), initial NIHSS score were significantly higher in insufficient medication group (6.1 ± 6.5 *VS* 4.4 ± 4.4, *P* = .004 for LAA; 3.8 ± 3.5 *VS* 2.7 ± 1.8, *P* = .014 for SVO). In multiple linear regression model, insufficient medication to antihypertensives had a significant effect on NIHSS score (*t* = 3.417, *P* = .001) after adjusting covariates.

**Conclusion:**

Insufficient medication of antihypertensives before stroke was independently associated with the severity of acute ischemic stroke. Further studies with prospective designs are warranted to evaluate clinical implication of adherence to antihypertensives for ischemic stroke.

## Background

Benefits of blood pressure lowering for primary stroke risk reduction are broadly consistent across a range of different population [1–4]. Recent studies support that more intensive control of blood pressure reduces risk of stroke more than less intensive control [5–8]. However, high adherence to antihypertensive treatment in terms of reduction of cardiovascular events has been relatively underscored in the field of evidence-based therapies of population [9].

Adherence to antihypertensive is closely related to the time within therapeutic range, and may influence on the cardiovascular prognosis [10]. The associations between non-adherence and stroke events followed a dose-response pattern, and the poorer the adherence, the greater the risk of death and hospitalization due to stroke [11]. The World Health Organization (WHO) had estimated that between 20–80 % of patients receiving treatment for hypertension are adherent [12]. In Korea, among people aged more than 30 years with hypertension, 61.9 % were on regular treatment, and only 43.6 % had their blood pressure under control [13]. Nonadherence to antihypertensive treatment is a common problem for cardiovascular prevention in Korean population.

The initial stroke severity has been reported as the most important predictor for poststroke outcome [14–16], and is correlated with stroke subtypes [17]. A few study reported a controversial class effect of antihypertensive drugs [18, 19] and the combination with antiplatelet or statin on stroke severity [20], but the relationship between the pre-stroke non-adherence to antihypertensives and stroke severity has been largely unknown.

This study aimed to investigate whether nonadherence to antihypertensives prior to acute ischemic stroke is associated with initial stroke severity in patients with hypertension.

## Methods

### Patients

A total 2335 consecutive patients with first acute (<7 days) ischemic stroke or imaging-positive transient ischemic attack were registered in Soonchunhyang University Hospital from Mar 2005 through Aug 2014. This web-based registry contains clinical characteristics, laboratory/imaging findings, and cardiovascular outcomes after discharge [21]. The study design was approved by the Soonchunhyang institutional review board before investigation. Hypertension was detected in 1660 (71.1 %) patients. The diagnostic criteria of hypertension were 1) history of diagnosis prior to acute ischemic stroke or 2) systolic/diastolic blood pressure (SBP/DBP) of ≥160/95 mmHg in at least two subsequent measurement after stable state of acute stroke or 3) evidence of left ventricular hypertrophy in echocardiography or hypertensive retinopathy during hospitalization. LVH with diabetes or other metabolic causes was excluded in case of no history of hypertension.

### Data collection and workup

Data were prospectively collected including history of hypertension, medication history, vascular risk factors, laboratory findings, stroke subtypes, initial National Institute of Health Stroke Scale (NIHSS) Score. Adherence to antihypertensives during the last one month were captured by patient’s or caregiver’s information, and were categorized into two groups: adherence (regular medication) and insufficient medication (diagnosed but no medication, irregular medication, and undetected before index stroke). The patients who took medications more than 80 % of the last one month were categorized as regular medication. The cases with missing data for adherence to antihypertensives prior to acute ischemic stroke were excluded (*n* = 93, 5.6 %).

Stroke subtypes were determined by extensive workups including magnetic resonance imaging/angiography, echocardiography, and Holter monitoring in all patients: large artery atherosclerosis (LAA), small vessel occlusion (SVO), cardioembolism (CE), and undetermined etiology (SUE). Stroke with other determined etiology were excluded (*n* = 29). Finally, 1538 cases were analyzed.

### Statistical analysis

Categorical variables were reported as frequency and continuous variables as mean ± standard deviation. Chi-square test was used to determine the relationship with categorical variables, and student T-test to compare the means of continuous variables between adherence and insufficient medication group. The difference of NIHSS score between adherence group and insufficient medication group were determined by T-test in each stroke subtype. Multiple linear regression model was established for initial NIHSS score adjusting covariates which have an influence on initial stroke severity in univariate analysis and previous reports. Statistical significance was defined as *P* <0.01 in multiple linear regression. All statistical analyses were done with SPSS 18.0 for Windows (Chicago, USA).

## Results

The patients who had taken antihypertensives regularly before stroke (adherence group) were 1232 (80.1 %). Insufficient medication group had 306 (19.9 %) patients: diagnosed but no medication (*n* = 54), irregular medication before acute stroke (*n* = 164), and diagnosed at admission (*n* = 88). The average age were 69.6 ± 11.5 in adherence group while those in insufficient medication group were 66.1 ± 12.2 (*P* < .001). The fraction of insufficient medication group were larger in female than male (22.1 % *VS* 18.1 %, *P* = .054). Insufficient medication group had higher initial NIHSS score than adherence group (6.5 ± 7.2 *VS* 5.4 ± 5.7, *P* = .11). The baseline characteristics between adherence and insufficient medication group were depicted in Table 1.

**Table 1:** Baseline characteristics between adherence and insufficient medication group

Characteristics	Adherence (*n* = 1232, 80.1 %)	Insufficient medication (*n* = 306, 19.9 %)	*P*
Age, years	69.6 ± 11.5	66.1 ± 12.2	<.001
Sex, male	704 (57.1 %)	156 (51.0 %)	.054
BMI (kg/m^2^)	24.0 ± 3.3	24.5 ± 3.6	.043
NIHSS, admission	5.4 ± 5.7	6.5 ± 7.2	.011
Initial SBP, mmHg	146.6 ± 26.4	151.6 ± 28.2	.004
Initial DBP, mmHg	85.8 ± 14.3	91.0 ± 15.6	<.001
Previous stroke history	284 (23.1 %)	57 (18.6 %)	.106
Previous antiplatelet	103 (8.7 %)	17 (5.6 %)	.121
Previous statins	97 (7.9 %)	15 (4.9 %)	.087
Antihypertensive class			
CCB	538 (43.7 %)	61 (37.2 %)^a^	.135
BB	271 (22.0 %)	33 (20.1 %)	.664
ARB	406 (33.0 %)	52 (31.7 %)	.823
ACEI	117 (9.5 %)	23 (14.0 %)	.103
Diuretics	303 (24.6 %)	34 (20.7 %)	.323
Diabetes	546 (44.3 %)	133 (43.5 %)	.839
Hyperlipidemia	302 (24.5 %)	152 (49.7 %)	<.001
Current smoking	256 (20.8 %)	103 (33.7 %)	<.001
Atrial fibrillation	323 (26.2 %)	48 (15.7 %)	<.001
Total cholesterol, mg/dL	167.0 ± 42.4	216.1 ± 44.2	<.001
Initial glucose, mg/dL	133.5 ± 60.5	141.0 ± 60.0	.082
Hemoglobin A1c, %	6.6 ± 1.5	6.9 ± 1.9	.011

Data are n (%) or mean ± SD values. *P* values for T-test and chi-square test

*SBP* systolic blood pressure, *DBP* diastolic blood pressure, *CCB* calcium channel blocker, *BB* beta blocker, *ARB* angiotensin receptor blocker, *ACEI* angiotensin converting enzyme inhibitor

^a^Denominator: nonadherent cases except unrecognized or no medication

Mean NIHSS scores among stroke subtypes were compared in Table 2. Cases with missing data for TOAST classification were not included (*n* = 17). NIHSS score were significantly higher in insufficient medication group in LAA and SVO: 6.1 ± 6.5 *VS* 4.4 ± 4.4, *P* = .004 for LAA; 3.8 ± 3.5 *VS* 2.7 ± 1.8, *P* = .014 for SVO. There was a tendency of higher NIHSS score of insufficient medication group in CE (12.7 ± 11.0 *VS* 9.3 ± 7.4, *P* = .141) and SUE (8.4 ± 8.3 *VS* 7.0 ± 6.8, *P* = .004).

**Table 2:** Mean NIHSS score among stroke subtypes

Stroke subtypes	Case	Adherence	Insufficient medication	*P*
Large artery atherosclerosis	731	4.4 ± 4.4	6.1 ± 6.5	.004*
Small vessel occlusion	260	2.7 ± 1.8	3.8 ± 3.5	.014*
Cardioembolism	214	9.3 ± 7.4	12.7 ± 11.0	.141
Undetermined etiology	316	7.0 ± 6.8	8.4 ± 8.3	.194
Total	1521	5.5 ± 5.7	6.5 ± 7.2	.017

Data are mean ± SD values. *P* values for T-test within each stroke subtype

*P < .05

In multiple linear regression model, R square was 0.653, and residual was independent (Durbin Watson = 1.813). The fitness of regression model was good (*F* = 18.716, *P* < .001). The strongest factor which influence on NIHSS score was atrial fibrillation (*t* = 10.944, *P* < .001). Age (*t* = 3.266, *P* < .001), initial systolic blood pressure (*t* = 3.396, *P* = .001) and previous stroke history (*t* = 2.717, *P* = .007) were also significant. Insufficient medication to antihypertensives had an independent effect on NIHSS score (*t* = 3.417, *P* = .001) (Table 3).

**Table 3:** Multiple linear regression model for initial NIHSS in acute ischemic stroke

Dependent variable	Independent variable	B	β	t	*P*-value	VIF
NIHSS	Constant	−2.23				
	Age	.050	.097	3.266	.001*	1.292
	Male	−.872	−.071	−2.428	.015	1.258
	Body Mass Index	−.051	−.028	−1.050	.294	1.050
	Previous stroke history	1.056	.073	2.717	.007*	1.052
	Atrial fibrillation	4.197	.294	10.944	<.001*	1.065
	Current smoking	.953	.067	2.255	.024	1.320
	Initial SBP	.021	.090	3.396	.001*	1.040
	Total cholesterol	.000	−.002	−.054	.957	1.278
	Hemoglobin A1c	.200	.053	2.019	.044	1.028
	Insufficient medication	1.489	.099	3.417	.001*	1.230

*P < .01

## Discussion

This study demonstrates that low antihypertensive medication adherence was related to severe acute ischemic stroke. Nonadherence is an important and very amenable factor to change among cardiovascular risk factors for hypertensive patients [22]. Self-reported adherence to cardiovascular medications in patients who have coronary artery disease is less than 40 % [23]. However, the nonadherence to antihypertensive for primary cardiovascular prevention would be as high as 50 % [24]. The efficacy of antihypertensives has been evaluated in randomized clinical trials (RCTs), but in actual practice, many patients who would be excluded from RCTs receive medication for a long time, and may not be as adherent with medication as those included in RCTs [25]. A tertiary medical center in Korea reported that average non-compliance to the single drug antihypertensive regimen was 16 % in the cardiology practice [26]. In general Korean population, however, about one third are not on regular antihypertensive treatment [13]. The overall rate of nonadherence was about 20 % in our 10-year observation study, and the fraction of nonadherence was higher in female.

Assessing adherence to hypertensive therapy for cardiovascular prevention is a complex process influenced by multiple factors [27]. Previous studies differentiates between adherent and nonadherent patients by prescription claim, drug refill, patients’ records, motivation and knowledge [23, 28, 29]. This study collected data about the regularity of medication by patient’s or caregiver’s description during the last one month before acute ischemic stroke. Given an observational nature of our study, this would lead to many epidemiological bias. However, because complicated scales could not be applied in the setting of acute stroke enrolling consecutive patients, we have used simple item to evaluate adherence before stroke in registry system. Although unrecognized hypertension should be considered separately from nonadherence to antihypertensives, this study was to measure stroke severity in whom blood pressure were not adequately controlled. Therefore, hypertension diagnosed at admission were included within insufficient medication group. Most previous study obtained the adherence data at one point of several years before stroke event [9, 11, 30], out study checked shortly before stroke reflecting recent adherence.

The pathophysilogic mechanisms between uncontrolled hypertension and severe stroke needs to be studied. One previous study suggested that prestroke beta-blocker use is related to less severe ischemic stroke by sympatholytic effect [18]. High blood pressure can result in increased glucocorticoid level and inflammatory marker which can aggravate cell death in penumbra area [31–34]. Reduction in arterial pressure by hydralazine with hydrochlorothiazide or an angiotensin converting enzyme inhibitor is protective against focal cerebral ischemia in stroke-prone spontaneously hypertensive rats model [35]. Nonadherence to antihypertensive medication prior to first stroke was associated with a 5.7-fold increased odds of fatal stroke during the year of death and a two-fold increased risk of nonfatal stroke [11]. Higher odds of fatal stroke than that of nonfatal stroke may indicate that nonadherence is closely related to more severe stroke.

It is noteworthy that the achievement of target BP goal is an important factor for preventing stroke. Although there was a significant difference in the stroke severity between adherence and insufficient medication groups, mean SBP/DBP were higher in insufficient medication group. The values of mean SBP/DBP which failed to reach a target goal may influence on the severity of acute ischemic stroke. However, the acute hypertensive response is very common in acute stroke period (occurring in >75 % of cases), and may be induced by increased sympathoadrenal tone, direct brain injury, systemic condition or stress response [36]. The difference of SBP/DBP can be a consequence of more severe stroke in insufficient group. For this reason a strict definition should be needed for the “history of hypertension” in acute stroke registry. The definition of hypertension in this study was 160/95 instead of 140/90. In the Framingham cohort, the “definite hypertension” was defined as 160/95 [37]. Previous studies assessed the history of stage II hypertension as blood pressure ≥160/95 mm Hg or use of antihypertensive medicine at baseline questionnaire [38].

Our study has several limitations. First, the data of insufficient medication were captured only for antihypertensives, but not for antiplatlets, antidiabetics or lipid lowering agents. Prestroke antiplatelets may be associated with stroke severity [39, 40]. However, the proportion of antiplatelet user before stroke was neither high nor different between the two groups in our study population (8.7 % *VS* 5.6 %, *P* = .121). Moreover, individual adherences to medications were not different across vascular risk factors [19]. Second, this study was single center-based, although the number of subjects was enough to be compared with previous studies. Our results need external validation to confirm the association between insufficient medication and stroke severity. Third, it is known that the NIHSS score does not generally show normal distribution to be an outcome variable in linear regression model. However, the case number of our study was sufficiently large with good explanation power and model fitness (R square = 0.653; *F* = 18.716, *P* < .001), and there was no multicollinearity between variables in our regression model (VIF <2). Finally, in our study, insufficient medication patients had more smokers and higher serum cholesterol level. Although the association between insufficient medication and stroke severity was significant after adjusting these variables, the social habit and healthy conditions should be considered as influencing factors on nonadherence in Korean.

## Conclusion

In conclusion, our study in the patients with acute ischemic stroke, insufficient medication to antihypertensives before stroke was independently associated with stroke severity. Further prospective cohort are warranted to evaluate clinical implication of adherence to medications for prevention of cardiovascular disease.
